# Classifying the Topology of AHL-Driven Quorum Sensing Circuits in Proteobacterial Genomes

**DOI:** 10.3390/s120505432

**Published:** 2012-04-27

**Authors:** Zsolt Gelencsér, Kumari Sonal Choudhary, Bruna Goncalves Coutinho, Sanjarbek Hudaiberdiev, Borisz Galbáts, Vittorio Venturi, Sándor Pongor

**Affiliations:** 1 Faculty of Information Technology, PázmányPéter Catholic University, Práter u. 50/a, Budapest 1083, Hungary; E-Mails: gelzsolt@gmail.com (Z.G.); galbo@digitus.itk.ppke.hu (B.G.); 2 International Centre for Genetic Engineering and Biotechnology (ICGEB), Padriciano 99, Trieste 32149, Italy; E-Mails: sonal.kumari@icgeb.org (K.S.C.); coutinho@icgeb.org (B.G.C.); sanjarbek.hudaiberdiev@icgeb.org (S.H.); 3 The Capes Foundation, Ministry of Education of Brazil, Cx postal 250, Brasilia, DF 70.040-020, Brazil; 4 Biological Research Center, Temesvári krt 62, Szeged 6726, Hungary

**Keywords:** quorum sensing, N-AHL, topology, proteobacteria, bacteria

## Abstract

Virulence and adaptability of many Gram-negative bacterial species are associated with an *N*-acylhomoserine lactone (AHL) gene regulation mechanism called quorum sensing (QS). The arrangement of quorum sensing genes is variable throughout bacterial genomes, although there are unifying themes that are common among the various topological arrangements. A bioinformatics survey of 1,403 complete bacterial genomes revealed characteristic gene topologies in 152 genomes that could be classified into 16 topological groups. We developed a concise notation for the patterns and show that the sequences of LuxR regulators and LuxI autoinducer synthase proteins cluster according to the topological patterns. The annotated topologies are deposited online with links to sequences and genome annotations at http://bacteria.itk.ppke.hu/QStopologies/.

## 1. Introduction

Bacteria might be simple, single celled organisms but their social behavior means they can form complex communities and engage in coordinated behaviors. Many bacterial species use chemical signals to monitor their environment and regulate population density. A pioneering study in bacterial chemical signaling was the unraveling of the regulation of luminescence in the marine bacterium *Vibrio fischeri* through a self-produced signal termed autoinducer [1]. *V. fischeri* regulates light production in the Hawaiian bobtail squid (*Euprymna scolopes*); when the population of *V. fischeri* reaches a certain density in the light organ of the squid, the concentration of the signaling molecule, exceeds a threshold extra-cellularly, triggering a cascade of cellular events that finally manifest in the production of light. More than 10 years later the signal was identified as an acyl homoserine lactone (AHL; [2]) and the genetic circuit was then also identified and shown to be composed of a signal generator called the *luxI*/LuxI gene/protein and a response-regulatory protein designated as *luxR*/LuxR gene/protein [3]. The name quorum sensing (QS) was then coined in the 1990s [4] to denote this cell-cell communication mechanism which is now recognized as a key trait governing bacterial community behavior [5–7]. In this mechanism, chemical signals trigger cellular actions such as cell division, the production of virulence factors as well as other traits that aid survival. Cross-talk between bacterial species may also determine survival in complex microbiota. Even inter-kingdom communication routes have been identified, including bacterial-fungal and plant-bacterium interactions [8,9]. The importance of quorum sensing has been clear for a number of years as it appears to be a promising target for controlling bacterial colonization and pathogenesis in human disease [10]. This could be of particular importance, as multidrug resistant strains of human pathogens continue to emerge. Potentially beneficial effects in relation to plant health can be attributed to QS in certain species including regulation of the production of anti-microbial compounds and induction of systemic resistance in plants [11].

One of the most well studied cellular and genetic mechanisms of QS is based on the *N*-acylhomoserine lactones (N-AHLs) [4]. Bacterial cells secrete and respond to autoinducers continuously to sense the surrounding environment, and respond to events as they occur. When an autoinducer reaches a critical level, the population of bacteria responds through a coordinated expression of specific target genes, which finally manifest in a particular behavior/phenotype. The N-AHL QS system is based on two proteins belonging to the LuxI and LuxR families [5,12]. Luxl-family proteins are cytoplasmic enzymes and are responsible for N-AHL synthesis [13]. After synthesis, the signal molecule moves freely across cell membranes and accumulates both intra- and extra-cellularly in proportion to cell density. When populations are low, N-AHL dissipates; when populations are high, N-AHL concentration increases. Above a critical concentration or cell density, N-AHLs interact with the LuxR-family [14] which in most cases result in complexes (homodimers) that bind specific promoter DNA sequences (termed *lux*-boxes) located in the promoter region of target QS-regulated genes ([15]. This subsequently affects their expression resulting in particular phenotypes of the organism. Most often one of the targets of AHL QS is the *luxI*-family gene resulting in a positive feedback loop.

As sequencing technologies have improved and many more bacterial genomes have been sequenced, it has become apparent that homologues of key *V. fischeri* N-AHL QS genes are present in the genomes of many Gram-negative bacteria [16,17]. N-AHLs are one of the most studied QS signaling molecules discovered so far; as of today over 70 species have been reported to possess N-AHL QS systems (see [17] for an earlier review). Most N-AHL producing bacteria possess canonical QS circuits that broadly resemble the original principles of the design that were described in the nineties. However, a number of interesting variations have since been described in the AHL synthesis/AHL response gene layout and regulation of the QS genes. The goal of this work is to show, based on a new survey of the genomic databases, how these variations correlate with the topological arrangement of the QS regulatory genes.

## 2. A Generalized Regulatory Framework of N-AHL-Based Quorum Sensing

**Figure 1.**
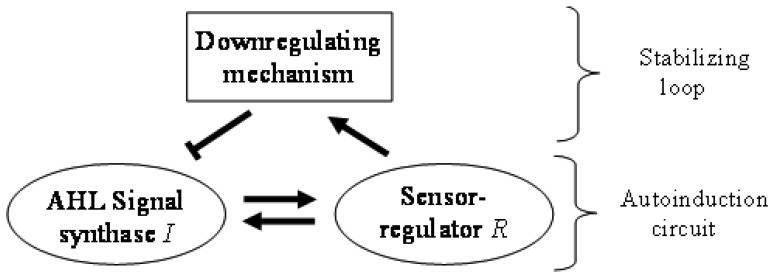
Regulatory outline of N-AHL-based QS signaling. Pointed arrows indicate activation, and the hammerhead arrow indicates inhibition.

In general terms, N-AHL-based QS signaling is often referred to as mere autoinduction, requiring only a synthase and a sensor/regulator protein. However, in the absence of regulation of QS, autoinduction would increase signal levels without limit. A down-regulation loop (Figure 1), which turns on at higher signal concentrations is the simplest way to limit and stabilize the signal levels. There are a variety of mechanisms that can play this role in QS systems. For example, the master regulator TraM can form a non-functional heterodimer with the TraR regulator in *Agrobacterium tumefaciens* [18,19]. The DNA-binding negative regulator RsaL acts as a homo-dimer by binding on the bi-directional *rsaL-luxI* promoter [20]. RsaM is another small regulatory protein believed to be acting in a similar way to RsaL in many *Burkholderia* species [21]. The crucial role of these negative regulators is highlighted by the fact that their deletion leads to signal overproduction and a less virulent bacterial phenotype. Bacterial genomes also encode enzymes that degrade AHLs in response to the stress signal ppGpp, which can also efficiently down-regulate QS signaling [22,23]. Down-regulation can also be achieved by RNA-based mechanisms. For example, some QS systems act by activating in their ground state an RNA-binding protein that inhibits transcription of synthases, and then gradually removing the inhibition when signal concentrations are increased [24,25]. Meanwhile, another class of QS regulation system is believed to use sRNA species that considerably decrease the mRNA of the LuxR genes at low population density [26–28]. For the sake of completeness we mention that downregulation can also be achieved by simple resource limitation where a reduction in number of QS cells would decrease of the QS signal concentration itself.

Regulatory circuits in which an element can both activate and inhibit another element are termed incoherent feed forward loops (IFFLs) [29,30]. In contrast to simple feed forward arrangements, IFFLs can exhibit a number of complex behavior patterns (for a review see [31]). While simple feed forward circuits have no inherent limits on their output, IFFL networks have bounded output, which ensures robustness against fluctuations in the input signal levels. The net result is the stabilization of the output signals mentioned above. Another noteworthy property of IFFL networks is adaptation: under appropriate circumstances the circuits will react to the change rather than to the level of the signal [32,33]. Theoretically, this property may also allow bacteria to react to changes rather than the level of the population density.

There are interesting variations also in the regulatory mechanism of the autoinduction loop itself. Although this mechanism is often thought to follow the *luxR/luxI* scenario in which the dimeric LuxR protein forms a regulatory complex with N-AHL, there are a growing number of cases where this happens in a different way. For example, a few members of the LuxR family are able to fold, dimerize, bind DNA, and regulate transcription in the absence of AHLs. Moreover, these proteins are antagonized by their cognate AHLs in the sense that some of the receptors/DNA complexes are disrupted by AHLs *in vitro* [34].

In the canonical AHL QS circuits the signal synthase is a LuxI type enzyme that synthesizes the signal molecule from S-adenosyl-methionine and an intra-cellularly synthesized acyl moiety delivered by an acyl carrier protein (ACP). However, more and more systems have been discovered where the acyl moiety is provided by acyl coenzyme A. Some of these systems use exogenous acyl (e.g., *p*-coumarate) moieties for signal synthesis, which provides a route to respond to environmental stimuli [35–38]. Characteristically, these non-canonical signal synthases tend to attach branched or non-aliphatic acyl side-chains.

## 3. Distribution and Frequency of QS Genes in Whole Bacterial Genomes

It was noted very early that *luxR* and *luxI* genes of the same QS circuit were most often located vicinal or very near to each other in the genomes. Other instances of *luxR* genes have also been identified in locations that are detached from QS circuits lacking a cognate N-AHL synthase. Such *luxR* genes are termed solos [9] or orphans [39] and are thought to allow bacteria to sense, among others, environmental stimuli and/or AHLs produced by neighboring bacteria. Case *et al.* analyzed the presence and absence of *luxR* and *luxI* genes in 512 genomes [17] in 2007 and found that such genes were exclusively present in proteobacterial genomes. Goryachev meanwhile, provided an excellent overview of the regulatory design principles of QS, distinguishing two chromosomal arrangements-one tandem *luxR/luxI* arrangement called type A, and one convergent arrangement called type B [40,41].

We recently started to survey the topological arrangements of N-AHL-based QS genes in pseudomonads [20]. In the present review we extend upon this work and survey the main categories of topological arrangements of QS genes in currently available bacterial genome sequences. We analyzed 1,403 complete genomes using standard bioinformatics methods (see Supplementary Materials for detailed methodology). This analysis was based on the reading frames annotated by the depositing authors and showed that 169 complete genomes contained QS genes that were close to other QS genes (*i.e.*, according to a distance threshold of 3,000 bp). All these were proteobacterial genomes. As the proteins encoded by *luxR* and *luxI* genes have spurious sequence similarities to a number of other protein families, we manually checked the length, sequence coverage and residue conservation of each similarity. We adopted this cautious approach in order to find a more reliable set of examples. For the same reason, draft genomes, solo *luxR* genes and other orphaned occurrences of QS genes were excluded from the detailed analysis. Unannotated genes were included only if they made part of a known topological arrangement. Of the 4.8 million genes we analyzed, 674 were *luxR* genes (unannotated: 33), 294 were *luxI* genes (unannotated: 13), 44 were rsa*L* genes (unannotated: 16) and 37 were rsa*M* (all were unannotated). The results of our search have been deposited to an open online repository from where the arrangements and the individual sequences can be retrieved (http://bacteria.itk.ppke.hu/QStopologies/).

It is a reasonable question to ask if the found occurrences reflect the frequency of QS genes in nature. We feel that this is not the case. First, we limited our analysis to cases where *luxR* and *luxI* genes are located near each other. Second, our analysis was based on similarity to known LuxR and LuxI proteins. Consequently, we left out lonely occurrences of luxR genes (solos), luxR genes that may control a different type of signal synthases, or signal synthases under the control of a different regulator protein. There were a few potential similarities outside the proteobacterial genomes, including the cyanobacterium *Gloeothece* PCC6909 which was suggested to have a QS system [42]. Nevertheless, we decided to limit our analysis to proteobacterial genomes where most of the well-established examples are found. Third, the analysis was limited to complete genomes, and this is a highly biased dataset. With these limitations we found QS genes in about 12% of complete proteobacterial genomes and it would be tempting to argue that this is in agreement with the expected frequency of AHL positive strains in proteobacteria (6–12%) [17]. At the moment we think however that this apparent agreement has to be confirmed with more rigorous sampling methods.

## 4. Topological Arrangements of QS Genes in Bacterial Genomes

Gene topology is a broad term that can include the arrangement of genes within chromosomes, with respect to the replication origin or other chromosomal elements. In this work we use the words “topological arrangement” or briefly “topology” to denote the arrangement within a close neighborhood of the QS regulatory genes, to denote whether the genes are convergent, divergent, synthase upstream, receptor upstream, or synthase and receptor separated by other genes, *etc*. The preliminary overview of the searches showed a variety of topological types. To illustrate these, we developed a concise notation based on a PROSITE-like syntax [43]. The *luxR, luxI, rsaL* and *rsaM* genes were abbreviated as *R, I, L* and *M*, respectively, and X is used for all other genes. An arrow above each gene symbol then shows the direction of transcription. With this notation, for example, *R⃗I⃗*denotes adjacent *luxR* and *luxI* genes transcribed in the same direction. *R⃗X*(>5)*I⃗* denotes the same pattern with more than five genes between the *luxR* and *luxI*, without specifying the direction of transcription of the X gene. A summary of the topological categories found in our survey is given in Table 1. Somewhat arbitrarily, we divided the patterns into two groups: simple topologies and complex topologies. Simple topologies consist of *luxI/luxR* genes that are either vicinal or are separated by few genes. Such arrangements are characterized by typical patterns of transcriptional orientation that are conserved in many proteobacteria. This category makes up the majority of the observed cases in Table 1 and is the main subject of this review. On the other hand, there are complex topologies in which the *luxR/luxI* genes are separated by a larger and more variable number of intervening genes. These topologies are characteristic of *Agrobacterium* and *Rhizobium* species. As there are many excellent reviews on these species [44,45], we mention this class only for the sake of completeness.

Within the class of simple topologies, the majority of the cases are made up of the *R⃗I⃗* and the *R⃗I⃐* topologies that Goryachev termed type A and type B, respectively [40,41]. In Table 1 we see combinations that are outside these two known categories. For instance, all four arrangements that are possible for two vicinal genes appear. In a characteristic subgroup of the simple patterns there is a single intervening gene between the *luxR* and *luxI* genes. These intervening genes show interesting commonalities in terms of function (Table 2). Out of 48 such single intervening genes 11 code RsaL and 29 code RsaM proteins that are both known to negatively regulate quorum sensing. In both cases of these proteins, typical topologies were observed: *R⃗L⃐I⃗* and *R⃐M⃗I⃗*, respectively. RsaL (*L* in our notation), is a member of the tetra helical superclass of H-T-H proteins [46] which are recognized as widespread QS repressors in bacteria, binding to DNA as dimers. For example, RsaL (which is predominant in pseudomonad genomes [47]) in *P. aeruginosa* prevents expression of the *R* gene by binding to DNA next to the *lux*-box [21]. In our analysis we also found that homologues of RsaL frequently occur outside QS circuits in various bacterial genomes (data not shown). The significance of this is not clear at this stage. In contrast, we found that RsaM (*M* in our notation), a protein of unknown structure that negatively regulates QS in *P. fuscovaginae* [21], seems to only occur in the context of QS circuits (see also [48]). Even though RsaM seems to occur mainly in the *R⃐M⃗I⃗* arrangement, it also appears in a number of other topologies (M1, M2 and M3; Table 1). Of the rest of the X genes found in RXI topology, *mupX* of *P. fluorescens* NCIMB10586 is an amidase-hydrolase, which is able to digest/degrade the AHL signal of the same species [49]. MupX can therefore also be considered as a negative QS regulator. From the rest of the X genes, two are involved in DNA-mobilization (an integrase and a transposase), and the rest are hypothetical proteins of unknown function.

Conserved gene overlaps are another commonality observed in short, conserved topologies. In our analysis, two topologies were found to contain this feature, namely *R⃗L⃐I⃗* (L1) and the *R⃗I⃐* (R2), which occurred in 15 and 53 proteobacterial genomes respectively. The L1 type QS circuits contain an overlap between transiently transcribed *luxR* and *rsaL* gene. The length of the overlap varies in different species. The L1 type QS circuit of *P. aeruginosa* contains an overlap of 10 bp and the same overlap is 20 bp long in *P. fuscovaginae*. In contrast, *P. putida* has an L1 circuit where the R and L are close (4 bp apart) but not overlapping [48]. The R2 topology contains an overlap of 2 to 79 bp between the convergently transcribed *luxR* and *luxI* genes. Previously it has been suggested that the expression of one member of a convergent or overlapping gene pair might antagonize the expression of the second member resulting in activation or repression of different functions or phenotypes [34]. This may happen by either failed recognition by the second RNA polymerase molecule, or hybridization of the two complimentary mRNAs, although this is yet to be confirmed. Even though overlapping genes are not uncommon in tightly co-regulated gene circuits of bacteria [50], e.g., restriction modification systems [36], the overlaps of convergently transcribed genes are less common in the literature. However, a few examples of convergent antisense RNA regulation have been reported also in bacteria, including S-box transcripts that are convergent to the *ubiG-mccBA* operon in *C. acetobutylicum* [51].

In contrast to simple topologies, the group of longer, complex topological patterns shows greater variety although the number of occurrences we found was considerably less. The groups of *Agrobacterium* and various *Rhizobia* often contain many genes between the *luxR* and *luxI* type genes. An interesting example in this group is the MI pattern of *B. ambifaria*, where two well distinguishable QS genes (*rsaM* and *luxI*) appear in their usual tandem topology, but without an annotated or computationally identifiable *luxR* homolog in their vicinity.

## 5. Taxonomic Distribution of QS Gene Topology Patterns

The number of complete genomes does not allow us yet to draw definitive conclusions about the preference of certain patterns to appear in various classes or species of bacteria. It is apparent however that the *R⃗I⃗* topology is predominant in α-proteobacteria, while the *R⃗I⃐* pattern is more frequent in γ-proteobacteria. Furthermore, the *R⃗L⃐I⃗* and the *R⃐M⃗I⃗* topologies seem to occur in the β and γ classes but not in α-proteobacteria. The question arises whether the known QS proteins, such as LuxI and LuxR, cluster simply according to the known taxonomy or rather according to the topological pattern. Cladograms of 154 LuxI and LuxR protein sequences (Supplementary Figures 3 and 4) show a clear tendency for clustering according to topological patterns. For instance, the *R⃗L⃐I⃗, R⃐M⃗I⃗, R⃗I⃗* and *R⃗L⃐* patterns form clearly distinguishable groups both in the LuxR and in the LuxI trees. Also, if two LuxI (or LuxR) proteins from the same topology group are nearest neighbors within the tree, they are very rarely from the same genome. In more detail, of the LuxI-type proteins that occur in genomes with multiple QS circuits, 38% have a nearest sequence similarity neighbor from the same family, 35% from the same genus, 26% from the same species, and approximately 1% from the same genome. The frequency with which *luxR* genes occur is similar: 37%, 36%, 27%, and 1%, respectively. This indicates that the patterns may have formed before the modern strains diverged from each other.

**Figure 2.**
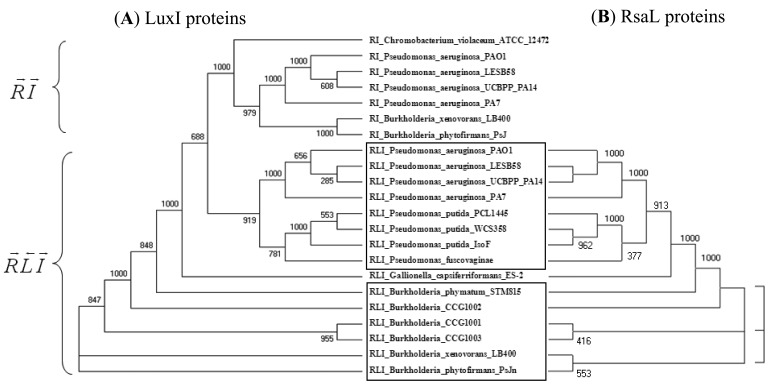
Clustering of LuxI and their negative regulators RsaL shows identical tendencies. (**A**) shows tree of LuxI proteins present RI and RLI pattern, and (**B**) shows RsaL proteins in RLI pattern. The sequences were taken from genomes harboring *rsaL* genes located between *luxR* and *luxI* genes. The clustering was carried out by the Phylip program package [52]. The numerical value at each node indicates the bootstrap value supporting every split in the lineage (out of 1,000 bootstrap replicates).

An example in Figure 2 shows that LuxI proteins present in *R⃗L⃐I⃗* patterns clearly separate from those present in *R⃗I⃗* patterns within the same genome and cluster together with the respective genes of another (β or γ) class (Figure 2(A)). At the same time, the clustering of the RsaL proteins (Figure 2(B)) is identical to the clustering of their accompanying LuxI genes (Figure 2(A)). In other words, QS proteins seem to cluster according to gene topology at various taxonomic levels, which suggests that the *luxR, luxI* as well as the intervening genes may have evolved together. Gene neighborhoods are known to evolve via complex rearrangements, with different combinations of genes from a neighborhood fixed in different lineages [53,54]. However, when the genes are part of fine-tuned regulatory networks, such as QS circuits, strict constraints may be imposed on the process of rearrangement.

## 6. Conclusions and Open Problems

We can conclude that the topological patterns in QS genes seem to follow a few basic rules—such as the conservation of genes between luxR and luxI genes, repeating patterns in certain taxa, *etc.*—but their variety is apparently greater than previously indicated [35,36]. The clustering of QS genes suggests that the topological units might act as regulatory modules that evolve together. In order to further test these hypotheses, we plan to widen the scope of the analyzed databases so as to include in the survey draft genomes and individually sequenced genomic regions. We also plan to pinpoint conserved DNA motifs within the neighborhood of the QS circuits. Maintaining and manually curating such a collection is beyond the possibilities of a single research group. Rather, we consider using bioinformatic tools for updating the current collection in the form of an openly accessible Internet repository so that researchers of the field could add their own annotations in the future.

This work is a preliminary, computational census of topological patterns found in complete proteobacterial genomes. It is expected that other kind of patterns may be found in published papers and in other sources, especially draft genomes, and we plan to develop computational protocols for finding these occurrences. On the other hand, there are a number of problems that are not touched upon in our work but may be answered as more detailed data become available. First, are the topological patterns associated with distinct biological functions? As QS circuits are involved in activating a large variety of genes in various bacteria, we speculate that there may be no simple correspondence between topology and the regulated functions. While we agree that these questions should be tackled by experiment, some supporting evidence can be gathered by comparing the gene neighborhood of QS circuits. The intervening genes (which are a conspicuous subgroup of neighborhood genes) appear to belong to only a few types, and in some genomes they are also found outside the immediate vicinity of QS genes. Another avenue would be to categorize the QS patterns according to the chemical nature of the signals produced by the *luxI* and/or sensed by the *luxR* gene.

The present classification suggests that the patterns seem to be relatively conserved and their distribution among the various taxonomic groups is not random. This does not mean that one can make broad statements about the functional reasons of this apparent conservation. We tend to speculate that the known principles of gene expression (co-transcription, repression by proteins or RNA) can be combined into a finite set of topological patterns that allow stable positive autoregulation and control of linked genes. It would be tempting to speculate that such modules then can move between genomes but this problem, as well as further evolutionary questions are outside the immediate scope of the present paper.

## Supplementary Material







## Figures and Tables

**Table 1.** Typical topological patterns found in complete bacterial genomes.

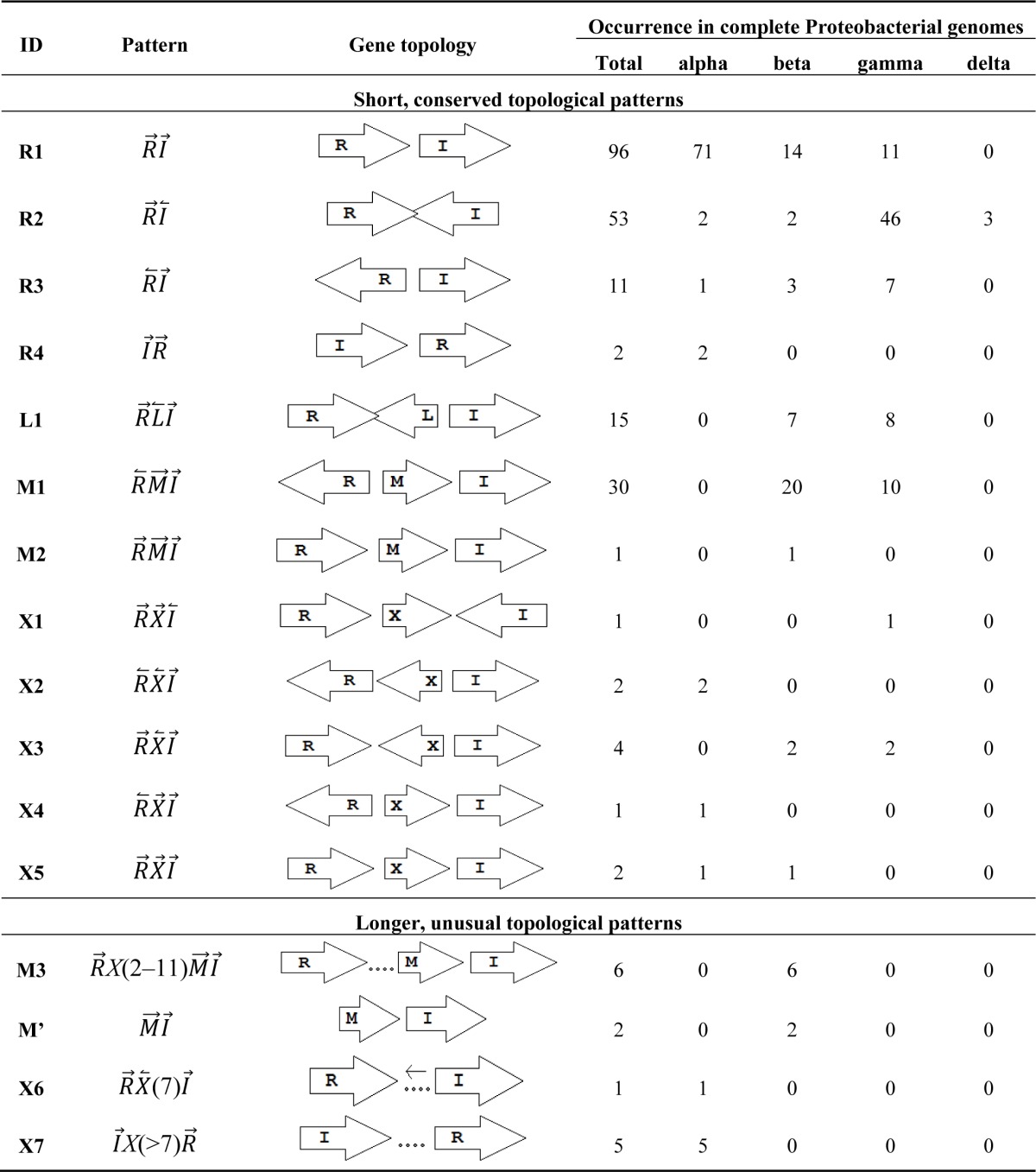

**Table 2.** Intervening genes in short, conserved topological patterns

**Gene-types**	**No. found in complete bacterial genomes**	**Potential role**	**Example of genomes**
*rsaL*	11	Negative regulator	*P. putida, P. fuscovaginae, P. aeruginosa* LESB58, *P. aeruginosa* PAO1
*rsaM*	29	Negative regulator	*B. pseudomallei* K96243
*mupX*	1	Negative regulator	*P. fluorescens NCIMB* 10586
Integrases/transposases	2	DNA mobilization	*B. vietnamiensis* G4 *Methylobacterium nodulans* ORS 2060
LuxR like regulator	1	?	*Gluconacetobacter diazotrophicus* PAl 5
Unknown function	5	?	*B. mallei* NCTC 10247, *Saccharophagus degradans* 2-40
