# Supplementary material for: Classifying the Topology of AHL-Driven Quorum Sensing Circuits in Proteobacterial Genomes
Source: Sensors (Basel). 2012 Apr 27;12(5):5432–44. doi: 10.3390/s120505432 (PMC3386692; doi:10.3390/s120505432)
Supplement: Supplementary file 1 [file sensors-12-05432-s001.pdf]

OPEN ACCESS

sensors

ISSN 1424-8220

www.mdpi.com/journal/sensors

Supplementary Information

Classifying the Topology of AHL-Driven Quorum Sensing Circuits in Proteobacterial Genomes. *Sensors* 2012, *12*, 5342-5444

Zsolt Gelencsér [1], Kumari Sonal Choudhary [2], Bruna Goncalves Coutinho [2,3], Sanjarbek Hudaiberdiev [2], Borisz Galbáts [1,4], Vittorio Venturi [2,*] and Sándor Pongor [2,*]

1 Faculty of Information Technology, PázmányPéter Catholic University, Práter u. 50/a, Budapest 1083, Hungary; E-Mails: gelzsolt@gmail.com (Z.G.); galbo@digitus.itk.ppke.hu (B.G.)

2 International Centre for Genetic Engineering and Biotechnology (ICGEB), Padriciano 99, Trieste 32149, Italy; E-Mails: sonal.kumari@icgeb.org (K.S.C.); coutinho@icgeb.org (B.G.C.); sanjarbek.hudaiberdiev@icgeb.org (S.H.)

3 The Capes Foundation, Ministry of Education of Brazil, Cx postal 250, Brasilia, DF 70.040-020, Brazil

4 Biological Research Center, Temesvári krt 62, Szeged 6726, Hungary

* Authors to whom correspondence should be addressed; E-Mails: venturi@icgeb.org (V.V.); pongor@icgeb.org (S.P.); Tel.: +39-040-3751; Fax: +39-040-226-555.

Zsolt Gelencsér *et al*. (2012): Classifying the topology of simple AHL-driven quorum sensing circuits in bacterial genomes" submitted to Sensors.

Data: Data of 1403 complete bacterial genomes were retrieved from the genome section of the NCBI (http://ncbi.nlm.nih.gov/). Additional protein sequences were retrieved from the UNIPROT database (http://www.uniprot.org/). Data retrieval was completed in December 15, 2011.

Methods: QS genes were determined using Hidden Markov Model (HMM) recognizers build with the HMMER programs HMMER 3.0, http://hmmer.janelia.org/). Briefly, a core set of protein sequences were taken for *LuxR*, *LuxI*, *rsaL* and *rsaM* proteins (see lists below). The CLUSTAL program (accessed via the EBI Webportal, http://www.ebi.ac.uk/Tools/msa/clustalw2/) was used for constructing the multiple sequence alignments which were then processed by the HMMBBUILD program to give HMM recognizers that were in turn used to scan the protein sequence data (over 4.7 million protein sequences). Sequences giving a E-value below 0.1 were manually checked for length, alignment coverage and residue conservation, and the topologies were assigned.

Protein sequences for selected sequence groups were downloaded from NCBI refseq database using a script which gave GIs as query and retrieved sequences in a file.

Cladograms for these selected sequence groups were built using the guide tree of the CLUSTAL program and visualized using the MEGA 5 program package installed from http://www.megasoftware.net.

The numerical value at each node indicates the bootstrap value supporting every split in the lineage (out of 1,000 bootstrap replicates).

Table 1. List of complete genomes in which QS genes were identified.

No	Species	Gene/protein id
1	Erwinia pyrifoliae Ep1/96	ERWPE
2	Pectobacterium wasabiae WPP163	PECWW
3	Halothiobacillus neapolitanus c2	HALNC
4	Edwardsiella tarda EIB202	EDWTE
5	Dickeya dadantii Ech586	DICD5
6	Burkholderia sp. CCGE1002	BURSC
7	Burkholderia sp. CCGE1001	9BURK
8	Citrobacter rodentium ICC168	CITRI
9	Rhodomicrobium vannielii ATCC 17100	RHOVT
10	Rhodopseudomonas palustris DX-1	RHOPX
11	Burkholderia sp. CCGE1003	BURSG
12	Pantoea ananatis LMG 20103	PANAM
13	Erwinia amylovora CFBP1430	ERWAC
14	Erwinia amylovora ATCC 49946	ERWAE
15	Sphingobium japonicum UT26S	SPHJU
16	Shewanella violacea DSS12	SHEVD
17	Yersinia pestis Z176003	YERPZ
18	Rhodobacter capsulatus SB 1003	RHOCB
19	Pantoea vagans C9-1	PANVC
20	Acinetobacter sp. DR1	ACISD
21	Ralstonia solanacearum PSI07	RALSL
22	Ralstonia solanacearum CFBP2957	RALSL
23	Erwinia billingiae Eb661	ERWBE
24	Gallionella capsiferriformans ES-2	GALCS
25	Dickeya dadantii 3937	DICD3
26	Asticcacaulis excentricus CB 48	ASTEC
27	Pantoea sp. At-9b	PANSA
28	Ralstonia solanacearum GMI1000	RALSO
29	Bradyrhizobium japonicum USDA 110	BRAJA
30	Mesorhizobium loti MAFF303099	RHILO
31	Sinorhizobium meliloti 1021	RHIME
32	Methylobacterium extorquens AM1	METEA
33	Yersinia pestis CO92	YERPE
34	Acidithiobacillus ferrooxidans ATCC 23270	ACIF2
35	Rhodospirillum rubrum ATCC 11170	RHORT
36	Burkholderia mallei ATCC 23344	BURMA
37	Burkholderia pseudomallei K96243	BURPS
38	Yersinia enterocolitica subsp. enterocolitica 8081	YERE8

Table 1. *Cont.*

No	Species	Gene/protein id
39	Burkholderia xenovorans LB400	BURXL
40	Ruegeria pomeroyi DSS-3	SILPO
41	Yersinia pestis KIM 10	YERPE
42	Saccharophagus degradans 2-40	SACD2
43	Pseudomonas syringae pv. syringae B728a	PSEU2
44	Pseudomonas aeruginosa PAO1	PSEAE
45	Burkholderia cenocepacia J2315	BURCJ
46	Rhizobium leguminosarum bv. viciae 3841	RHIL3
47	Pectobacterium atrosepticum SCRI1043	ERWCT
48	Pseudomonas syringae pv. tomato str. DC3000	PSESM
49	Pseudomonas aeruginosa UCBPP-PA14	PSEAB
50	Chromobacterium violaceum ATCC 12472	CHRVO
51	Yersinia pestis biovar Microtus str. 91001	YERPE
52	Chelativorans sp. BNC1	MESSB
53	Burkholderia sp. 383	BURS3
54	Burkholderia vietnamiensis G4	BURVG
55	Burkholderia thailandensis E264	BURTA
56	Pseudomonas syringae pv. phaseolicola 1448A	PSE14
57	Jannaschia sp. CCS1	JANSC
58	Yersinia pseudotuberculosis IP 32953	YERPS
59	Vibrio fischeri ES114	VIBF1
60	Paracoccus denitrificans PD1222	PARDP
61	Agrobacterium vitis S4	AGRVS
62	Agrobacterium radiobacter K84	AGRRK
63	Pseudoalteromonas atlantica T6c	PSEA6
64	Nitrobacter winogradskyi Nb-255	NITWN
65	Burkholderia ambifaria AMMD	BURCM
66	Sphingopyxis alaskensis RB2256	SPHAL
67	Burkholderia cenocepacia HI2424	BURCH
68	Burkholderia cenocepacia AU 1054	BURCA
69	Rhizobium etli CFN 42	RHIEC
70	Burkholderia mallei NCTC 10229	BURM9
71	Burkholderia mallei NCTC 10247	BURM7
72	Burkholderia mallei SAVP1	BURMS
73	Burkholderia pseudomallei 668	BURP6
74	Burkholderia pseudomallei 1710b	BURP1
75	Acidovorax citrulli AAC00-1	ACIAC
76	Rhodopseudomonas palustris HaA2	RHOP2
77	Rhodopseudomonas palustris BisB5	RHOPS
78	Rhodopseudomonas palustris BisB18	RHOPB
79	Rhodopseudomonas palustris BisA53	RHOP5
80	Geobacter uraniireducens Rf4	GEOUR

Table 1. *Cont.*

No	Species	Gene/protein id
81	Yersinia pestis Angola	YERPG
82	Yersinia pseudotuberculosis IP 31758	YERP3
83	Bradyrhizobium sp. BTAi1	BRASB
84	Burkholderia pseudomallei 1106a	BURP0
85	Geobacter sp. FRC-32	GEOSF
86	Sinorhizobium medicae WSM419	SINMW
87	Sodalis glossinidius str. morsitans	SODGM
88	Roseobacter denitrificans OCh 114	ROSDO
89	Yersinia pestis Antiqua	YERPA
90	Yersinia pestis Nepal516	YERPN
91	Acidithiobacillus ferrooxidans ATCC 53993	ACIF5
92	Aeromonas hydrophila subsp. hydrophila ATCC 7966	AERHH
93	Yersinia pestis Pestoides F	YERPP
94	Pseudomonas aeruginosa PA7	PSEA7
95	Aeromonas salmonicida subsp. salmonicida A449	AERS4
96	Burkholderia phymatum STM815	BURP8
97	Burkholderia ambifaria MC40-6	BURA4
98	Dinoroseobacter shibae DFL 12	DINSH
99	Shewanella woodyi ATCC 51908	SHEWM
100	Serratia proteamaculans 568	SERP5
101	Burkholderia phytofirmans PsJN	BURPP
102	Acinetobacter baumannii ATCC 17978	ACIBT
103	Acinetobacter baumannii ACICU	ACIBC
104	Burkholderia cenocepacia MC0-3	BURCC
105	Methylobacterium extorquens PA1	METEP
106	Methylobacterium sp. 4-46	METS4
107	Methylobacterium radiotolerans JCM 2831	METRJ
108	Vibrio fischeri MJ11	VIBFM
109	Burkholderia multivorans ATCC 17616	BURM1
110	Methylobacterium chloromethanicum CM4	METC4
111	Methylobacterium populi BJ001	METPB
112	Bradyrhizobium sp. ORS278	BRASO
113	Phenylobacterium zucineum HLK1	PHEZH
114	Rhizobium leguminosarum bv. trifolii WSM1325	RHILS
115	Rhodopseudomonas palustris TIE-1	RHOPT
116	Rhizobium leguminosarum bv. trifolii WSM2304	RHILW
117	Methylobacterium nodulans ORS 2060	METNO
118	Erwinia tasmaniensis Et1/99	ERWT9
119	Beijerinckia indica subsp. indica ATCC 9039	BEII9
120	Sinorhizobium fredii NGR234	RHISN
121	Acinetobacter baumannii AB0057	ACIB5
122	Rhizobium etli CIAT 652	RHIE6
123	Yersinia pseudotuberculosis YPIII	YERPY
124	Yersinia pseudotuberculosis PB1/+	YERPB

Table 1. *Cont.*

No	Species	Gene/protein id
125	Aliivibrio salmonicida LFI1238	ALISL
126	Acinetobacter baumannii AB307-0294	ACIB3
127	Pseudomonas aeruginosa LESB58	PSEA8
128	Candidatus Hamiltonella defensa 5AT (Acyrthosiphon pisum)	HAMD5
129	Pectobacterium carotovorum subsp. carotovorum PC1	PECCP
130	Dickeya zeae Ech1591	DICZE
131	Desulfovibrio magneticus RS-1	DESMR
132	Burkholderia glumae BGR1	BURGB
133	Edwardsiella ictaluri 93-146	EDWI9
134	Methylocella silvestris BL2	METSB
135	Variovorax paradoxus S110	VARPS
136	Ketogulonicigenium vulgare Y25	KETVY
137	Enterobacter cloacae SCF1	ENTCS
138	Gluconacetobacter diazotrophicus PAl 5	GLUDA
139	Methylobacterium extorquens DM4	METED
140	Acinetobacter baumannii AYE	ACIBY
141	Mesorhizobium ciceri biovar biserrulae WSM1271	MESCW
142	Rhodopseudomonas palustris CGA009	RHOPA
143	Collimonas fungivorans Ter331	COLFT
144	Cupriavidus necator N-1	CUPNE
145	Delftia sp. Cs1-4	DELSC
146	Mesorhizobium opportunistum WSM2075	MESOW
147	Novosphingobium sp. PP1Y	9SPHN
148	Roseobacter litoralis Och 149	ROSLO
149	Sinorhizobium meliloti AK83	SINMK
150	Sphingobium chlorophenolicum L-1	SPHCR
151	Sphingobium sp. SYK-6	9SPHN
152	Vibrio anguillarum 775	VIBAN

Table 2. Additional sequences from incomplete genomes, used in this study.

No.	Genome	DNA Ids	Protein ids
1	*P. fuscovaginae*	182375405, 290454884	182375406, 182375407, 182375408, 290454885, 290454886, 290454887
2	*P. putida* PCL1445	73672740	73672742, 73672743, 73672744
3	*P. putida* WCS358	45259341	45259342, 45259343, 45259344
4	*P. putida* IsoF	26417537	26417540,26417541, 26417542

Table 3. List of protein sequence sets used for building HMM recognizers.

No	Proteins	Gene/protein ids
	luxI homologues	
		YP_002649215.1, YP_003261728.1, YP_003262850.1, YP_003296640.1, YP_003331715.1, YP_003608088.1, YP_004230809.1, YP_003366470.1, YP_004012993.1, YP_004106681.1, YP_004106954.1, YP_004108425.1, YP_003910269.1, YP_003520250.1, YP_003530770.1, YP_003538486.1, YP_003546445.1, YP_003558209.1, YP_003566926.1, YP_003568278.1, YP_003576501.1, YP_003729883.1, YP_003930460.1, YP_003734012.1, YP_003749682.1, YP_003750860.1, YP_003744153.1, YP_003740503.1, YP_003740954.1, YP_003847234.1, YP_003885141.1, YP_004088230.1, YP_004115279.1, NP_521405.1, NP_522340.1, NP_767703.1, NP_106262.1, NP_106661.1, NP_109412.1, NP_385945.1, YP_002965845.1, YP_002966879.1, YP_002346031.1, YP_002347420.1, YP_002426405.1, YP_428477.1, YP_105963.1, YP_106161.1, YP_110894.1, YP_111576.1, YP_001005892.1, YP_554693.1, YP_555669.1, YP_165635.1, YP_167511.1, NP_669050.1, NP_670673.1, YP_528965.1, YP_234707.1, NP_250123.1, NP_252166.1, YP_002232872.1, YP_002234481.1, YP_768958.1, YP_048233.1, NP_793636.1, YP_789671.1, YP_791820.1, NP_903761.1, NP_993604.1, NP_994737.1, YP_674865.1, YP_371808.1, YP_001114940.1, YP_001117676.1, YP_439001.1, YP_439708.1, YP_273860.1, YP_508562.1, YP_071011.1, YP_071751.1, YP_206882.1, YP_914595.1, YP_002551489.1, YP_002549360.1, YP_002541324.1, YP_659946.1, YP_317245.1, YP_776005.1, YP_617566.1, YP_617628.1, YP_838353.1, YP_623506.1, YP_470411.1, YP_473057.1, YP_001024425.1, YP_001025818.1, YP_001077901.1, YP_001078152.1, YP_989942.1, YP_001062290.1, YP_001063210.1, YP_335777.1, YP_337633.1, YP_972130.1, YP_484039.1, YP_486927.1, YP_567542.1, YP_569311.1, YP_530592.1, YP_531903.1, YP_781244.1, YP_001231849.1, YP_001604809.1, YP_001606209.1, YP_001399709.1, YP_001400525.1, YP_001220569.1, YP_001241094.1, YP_001242901.1, YP_001075256.1, YP_001076162.1, YP_002537871.1, YP_001327237.1, YP_453964.1, YP_681952.1, YP_650194.1, YP_651865.1, YP_647981.1, YP_649109.1, YP_002220095.1, YP_855089.1, YP_001161918.1, YP_001163229.1, YP_001347034.1, YP_001349251.1, YP_001143471.1, YP_001583944.1, YP_001860597.1, YP_001811255.1, YP_001531662.1, YP_001534185.1, YP_001761364.1, YP_001476305.1, YP_001888022.1, YP_001893789.1, YP_001083198.1, YP_001844795.1, YP_001777918.1, YP_001779189.1, YP_001641952.1, YP_001772211.1, YP_001758390.1, YP_001776814.1, YP_001783295.1, YP_002158590.1, YP_001948920.1, YP_002423669.1, YP_001927659.1, YP_001203094.1, YP_002128524.1, YP_002976728.1, YP_001989358.1, YP_001991324.1, YP_002282165.1, YP_002495630.1, YP_002496260.1, YP_002497058.1, YP_001906897.1, YP_001908005.1, YP_001832057.1, YP_002826208.1, YP_002317565.1, YP_001979200.1, YP_001985290.1, YP_001719546.1, YP_001720402.1, YP_001873009.1, YP_001873806.1, YP_002265246.1, YP_002327281.1, YP_002439140.1, YP_002441565.1, YP_002923740.1, YP_003019698.1, YP_003002473.1, YP_002955226.1, YP_002909043.1, YP_002934276.1, YP_002360442.1, YP_002947663.1, YP_003964946.1, YP_003941574.1, YP_001603070.1, YP_003070966.1, YP_001715479.1, YP_004144716.1, YP_004145051.1, NP_945673.1
	LuxR homologues	
		YP_002649216.1, YP_003261727.1, YP_003262848.1, YP_003296639.1, YP_003331714.1, YP_003608086.1, YP_004230807.1, YP_003366469.1, YP_004012994.1, YP_004106680.1, YP_004106955.1, YP_004108424.1, YP_003910271.1, YP_003520251.1, YP_003530769.1, YP_003538485.1, YP_003546444.1, YP_003558208.1, YP_003566925.1, YP_003568279.1, YP_003576500.1, YP_003729882.1, YP_003930459.1, YP_003734010.1, YP_003749681.1, YP_003750859.1, YP_003744152.1, YP_003740504.1, YP_003740953.1, YP_003847232.1, YP_003885142.1, YP_004088229.1, YP_004115278.1, NP_521406.1, NP_522339.1, NP_767702.1, NP_106261.1, NP_106660.1, NP_109411.1, NP_385944.1, YP_002965846.1, YP_002966880.1, YP_002346032.1, YP_002347421.1, YP_002426403.1, YP_428476.1, YP_105961.1, YP_106160.1, YP_110896.1, YP_111575.1, YP_001005891.1, YP_554691.1, YP_555670.1, YP_165634.1, YP_167510.1, NP_669049.1, NP_670674.1, YP_528967.1, YP_234708.1, NP_250121.1, NP_252167.1, YP_002232873.1, YP_002234479.1, YP_768957.1, YP_048234.1, NP_793635.1, YP_789670.1, YP_791822.1, NP_903760.1, NP_993605.1, NP_994736.1, YP_674864.1, YP_371810.1, YP_001114942.1, YP_001117674.1, YP_439002.1, YP_439706.1, YP_273861.1, YP_508561.1, YP_071012.1, YP_071752.1, YP_206883.1, YP_914594.1, YP_002551488.1, YP_002549361.1, YP_002541325.1, YP_659944.1, YP_317246.1, YP_776003.1, YP_617565.1, YP_617627.1, YP_838351.1, YP_623508.1, YP_470410.1, YP_473056.1, YP_001024423.1, YP_001025820.1, YP_001077903.1, YP_001078154.1, YP_989940.1, YP_001062292.1, YP_001063209.1, YP_335776.1, YP_337635.1, YP_972129.1, YP_484040.1, YP_486928.1, YP_567541.1, YP_569310.1, YP_530593.1, YP_531902.1, YP_781245.1, YP_001231850.1, YP_001604810.1, YP_001606210.1, YP_001399708.1, YP_001400524.1, YP_001220570.1, YP_001241092.1, YP_001242900.1, YP_001075258.1, YP_001076161.1, YP_002537872.1, YP_001327236.1, YP_453965.1, YP_681951.1, YP_650193.1, YP_651866.1, YP_647982.1, YP_649110.1, YP_002220093.1, YP_855090.1, YP_001161917.1, YP_001163230.1, YP_001347033.1, YP_001349253.1, YP_001143472.1, YP_001583946.1, YP_001860599.1, YP_001811253.1, YP_001531661.1, YP_001534186.1, YP_001761363.1, YP_001476304.1, YP_001888024.1, YP_001893790.1, YP_001083200.1, YP_001844797.1, YP_001777917.1, YP_001779191.1, YP_001641953.1, YP_001772212.1, YP_001758389.1, YP_001776815.1, YP_001783296.1, YP_002158591.1, YP_001948918.1, YP_002423670.1, YP_001927660.1, YP_001203095.1, YP_002128523.1, YP_002976727.1, YP_001989359.1, YP_001991323.1, YP_002282164.1, YP_002495629.1, YP_002496262.1, YP_002497059.1, YP_001906896.1, YP_001908006.1, YP_001832058.1, YP_002826207.1, YP_002317567.1, YP_001979199.1, YP_001985289.1, YP_001719545.1, YP_001720401.1, YP_001873010.1, YP_001873807.1, YP_002265247.1, YP_002327279.1, YP_002439139.1, YP_002441567.1, YP_002923741.1, YP_003019697.1, YP_003002472.1, YP_002955225.1, YP_002909041.1, YP_002934275.1, YP_002360441.1, YP_002947664.1, YP_003964947.1, YP_003941575.1, YP_001603072.1, YP_003070967.1, YP_001715477.1, YP_004144717.1, YP_004145052.1, NP_945674.1,
	RsaL homologue	
		NP_250122.1, YP_001349252.1, YP_001860598.1, YP_001888023.1, YP_002441566.1, YP_002794907.1, YP_003608087.1, YP_003847233.1, YP_003910270.1, YP_554692.1, YP_791821.1
	RsaM homologues	
		YP_439707.1, YP_001062653.1, YP_776004.1, YP_001117675.1

© 2012 by the authors; licensee MDPI, Basel, Switzerland. This article is an open access article distributed under the terms and conditions of the Creative Commons Attribution license (http://creativecommons.org/licenses/by/3.0/).
