# Supplementary figures and images for: Classifying the Topology of AHL-Driven Quorum Sensing Circuits in Proteobacterial Genomes
Source: Sensors (Basel). 2012 Apr 27;12(5):5432–44. doi: 10.3390/s120505432 (PMC3386692; doi:10.3390/s120505432)

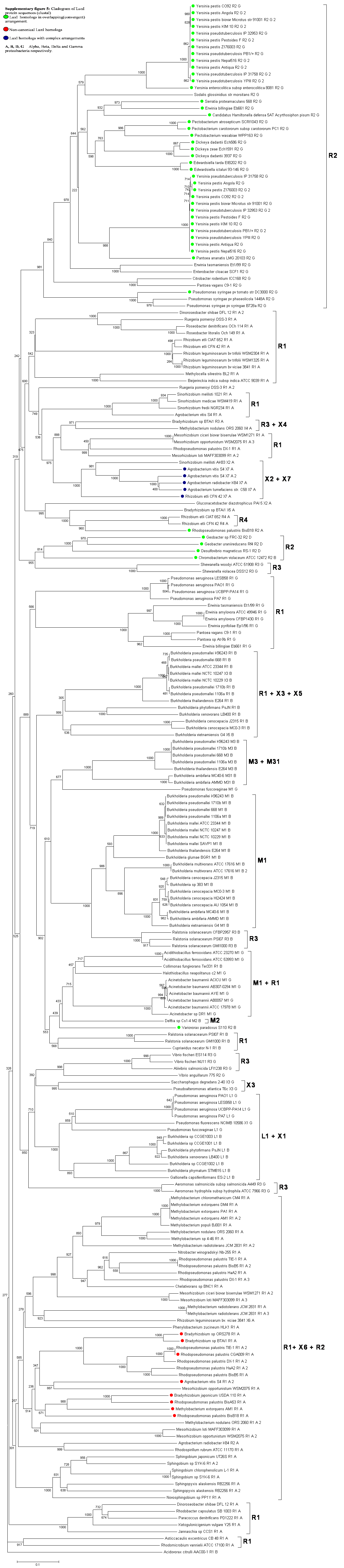

Supplement: Supplementary file 2 [file sensors-12-05432-s002.png]

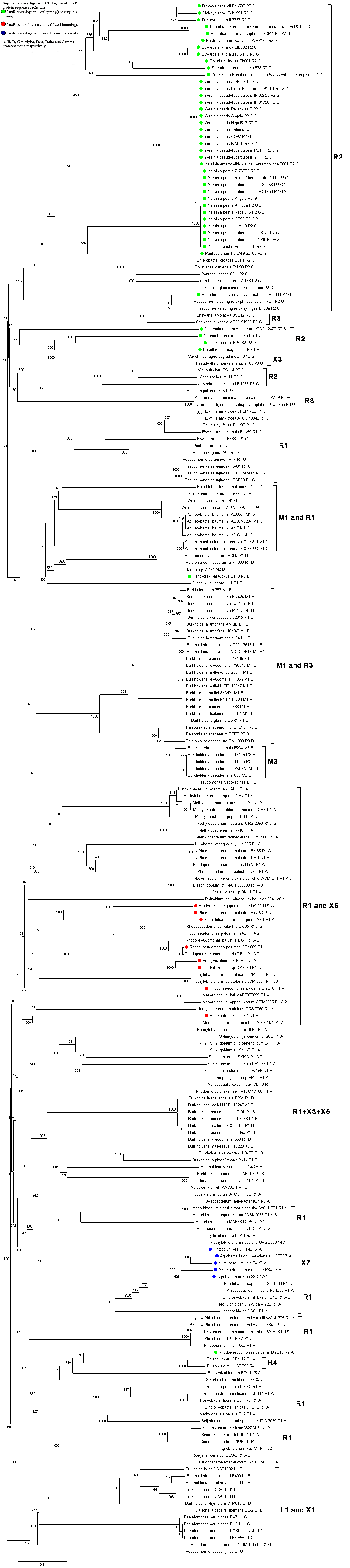

Supplement: Supplementary file 3 [file sensors-12-05432-s003.png]
